# Understanding the patient experience in hepatocellular carcinoma: a qualitative patient interview study

**DOI:** 10.1007/s11136-021-02903-4

**Published:** 2021-06-11

**Authors:** Nikunj Patel, Joshua Maher, Xandra Lie, Chad Gwaltney, Afsaneh Barzi, Mark Karwal, Teresa Macarulla, Hui-Chuan Sun, Jörg Trojan, Oren Meyers, Christina Workman, Shethah Morgan, Alejandra Negro, Gordon Cohen

**Affiliations:** 1 AstraZeneca, 1 Medimmune Way, Gaithersburg, MD 20878 USA; 2 IQVIA, Reading, UK; 3 IQVIA, Amsterdam, The Netherlands; 4 Gwaltney Consulting, Westerly, RI USA; 5 City of Hope Comprehensive Cancer Center, Duarte, Los Angeles, CA USA; 6 University of IOWA Health Care, Iowa City, IA USA; 7 GI Unit Vall D´Hebron Hospital, Barcelona, Spain; 8 Liver Cancer Institute, Zhongshan Hospital, Fudan University, Shanghai, China; 9 Medical Department 1, Goethe University Medical Center, Frankfurt, Germany; 10 IQVIA, New York, NY USA; 11 AstraZeneca, Cambridge, UK

**Keywords:** Hepatocellular carcinoma, Patient-reported outcomes, Qualitative, Quality of life, Symptoms

## Abstract

**Purpose:**

This study aimed to elucidate the patient experience of hepatocellular carcinoma (HCC) to guide patient-centered outcome measurement in drug development.

**Methods:**

Patients with HCC participated in qualitative interviews to elicit disease-related signs/symptoms and impacts, using discussion guides developed from literature searches and discussions with oncologists. Interview participants rated the disturbance of their experiences (0–10 scale). A conceptual model was developed and mapped against patient-reported outcome (PRO) instruments identified from database reviews.

**Results:**

Interviews were conducted with 25 individuals with HCC (68% were men; median age: 63 years; 12% Barcelona clinic liver cancer (BCLC) stage A; 32% stage B; and 56% stage C) in the USA. Fifty-one HCC-related concepts were identified from the interviews and were grouped into eight sign/symptom categories (eating behavior/weight changes; extremities [arms, legs]; fatigue and strength; gastrointestinal; pain; sensory; skin; other) and four impact categories (emotional; physical; cognitive function; other) for the conceptual model. The most prevalent and disturbing experiences across the disease stages were fatigue/lack of energy and emotional impacts such as frustration, fear, and depression. Abdominal pain and skin-related issues were particularly common and disturbing in individuals with HCC stage C. The EORTC QLQ-C30 and HCC18 were identified as commonly used PRO instruments in HCC studies and captured the relevant signs/symptoms associated with the patient experience.

**Conclusion:**

Patients with HCC reported a range of signs/symptoms and impacts that negatively affect daily functioning and quality of life. Including PRO measures in HCC clinical trials can provide meaningful patient perspectives during drug development.

**Supplementary Information:**

The online version contains supplementary material available at 10.1007/s11136-021-02903-4.

## Plain English Summary

Hepatocellular carcinoma (HCC) is the most common type of liver cancer. Little is known about the lived experience of people with HCC. People with HCC want to learn what to expect in the various stages of HCC and to have clearer expectations about HCC-related symptoms and impacts on daily functioning. Researchers need to know which symptoms and impacts relevant to patients to measure in clinical studies of new HCC treatments. Clinicians need to understand the effects of treatment on disease when making treatment decisions. In this study, we explored in depth the experiences of 25 people with HCC at various disease stages. We also assessed if symptoms relevant to people with HCC are included in a patient-completed questionnaire that is commonly used in HCC clinical studies: the European Organisation for Research and Treatment of Cancer Quality of Life Questionnaire and its HCC-specific module (EORTC QLQ-C30/QLQ-HCC18). People with HCC reported a range of symptoms and impacts that negatively affect their daily functioning and quality of life. The most prominent issues were fatigue, frustration, fear, and depression. People with advanced disease, which had spread from the liver or reoccurred, also reported abdominal pain and skin-related issues as particularly common and disturbing. The EORTC QLQ-C30/QLQ-HCC18 was shown to capture the relevant symptoms that are most meaningful to patients with HCC. Findings from this study provide much-needed information about people’s lived experiences of HCC. Our study findings support inclusion of the EORTC QLQ-C30/QLQ-HCC18 in clinical studies of new HCC treatments to capture meaningful patient perspectives.

## Background

The burden of liver cancer is high, with approximately 840,000 people worldwide receiving a diagnosis of liver cancer each year and 780,000 dying as a result of the disease [1]. Hepatocellular carcinoma (HCC) is the most common type of liver cancer, accounting for about 75–85% of cases [2, 3]. The prognosis for people with HCC is poor, with an estimated 5-year survival of only 12% following diagnosis [1, 3]. The most common risk factors for HCC are viral hepatitis B, viral hepatitis C, heavy alcohol consumption, and non-alcoholic fatty liver disease, the latter being driven by the increasing prevalence of obesity and type 2 diabetes mellitus [2–4]. The prevalence of underlying risk factors affects disease incidence, which is highest in East Asia, South-East Asia, and sub-Saharan Africa, primarily driven by geographic distribution of viral hepatitis prevalence and is higher in men than in women [2–4].

Novel approaches for HCC management, using targeted agents and immunotherapies administered as monotherapy [5, 6] or combination therapies [7–9], have experienced several advancements in recent years. There is a paucity of published data on the lived experience of patients with HCC. Only a few qualitative interview studies have elicited the signs, symptoms, and impacts of the disease directly from patients with HCC [10, 11]. A US study that included 10 patients with HCC (performance status ≤ 2) who had received systemic therapy identified diarrhea, fatigue, skin toxicities, and loss of appetite as patients’ most important concerns [11]. Pain, in particular abdominal pain, emerged as another important experience upon prompted questioning [11]. In a study from Taiwan, which included 33 patients who had received surgery, transarterial embolization/transarterial chemoembolization (TAE/TACE), or drug therapy for their HCC, both physical symptoms (e.g., digestive problems, sleep problems) and psychosocial stress were identified as being a significant part of the patient experience [10]. Interviews and pre-testing were also conducted with individuals with HCC as part of developing the HCC-specific module of the European Organisation for Research and Treatment of Cancer Quality of Life Questionnaire (EORTC QLQ-C30) [12], QLQ-HCC18 [13]. Most of the participants in that study had very early stage (Child–Pugh A) disease [13]. Fatigue, body image, and pain were among the issues considered important. The interview studies are informative, but there is still a limited understanding of the patient experience of HCC at different disease stages.

Evaluating patients’ experiences of their HCC in clinical trials is vital so that patients and clinicians can have clearer expectations regarding the effects of new treatments on symptoms and functional impacts that influence health-related quality of life (HRQoL). An in-depth understanding of these experiences is needed in order to select or to develop tailored measures of symptoms and impacts that can be used in HCC trials. This information is useful for regulators and payers, to guide decision making for product approval and reimbursement [14–18]. It is also an essential part of the development and testing of patient-reported outcome (PRO) instruments, to ensure that they are valid and fit for purpose in the intended target population [19–22]. PRO assessment should be included in clinical studies to provide direct evidence of treatment benefit from patients. PROs have become an important endpoint when evaluating the effectiveness of study treatments in clinical studies, and they aid the understanding of benefit-risk evaluation [23].

The aim of the current study was to obtain a detailed understanding of the patient experience of HCC-related signs or symptoms (signs/symptoms) and impacts on daily functioning and HRQoL.

## Methods

Qualitative concept elicitation interviews were conducted with individuals with HCC to identify the signs/symptoms and impacts of HCC that are most relevant to patients and to develop a model describing the concepts (symptoms and impacts) that people with HCC may experience [18, 20]. A qualitative content analysis method was used. Salient concepts from the interviews were mapped to PRO instruments frequently used in HCC studies.

### Patients and procedures

The qualitative patient interviews were conducted in accordance with recommendations provided by International Society for Pharmacoeconomics and Outcomes Research (ISPOR) Good Research Practices Task Force [20]. The study was approved by the New England Institutional Review Board.

Participants (aged ≥ 18 years) with confirmed diagnosis of early, intermediate, or advanced HCC (Barcelona clinic liver cancer (BCLC) stage A, B, or C) were recruited in the USA with the assistance of a patient advocacy group (Blue Faery), a clinical research organization (Renovatio Clinical), and a patient recruitment agency (Global Perspectives). Processes for recruitment included discussion boards and Facebook support groups (Blue Faery), email and social media outreach, and physician contact. Interview participants’ performance status, stage, and etiology of liver cirrhosis were not captured. All participants provided their written informed consent online before being invited for interviews and received a gift voucher (US$150) for their participation in the study. The qualitative interviews were held by telephone by three trained interviewers. All interviewers were educated to at least Bachelor’s level in life sciences subjects and were experienced in conducting individual patient concept elicitation interviews across a wide range of therapeutic areas. Participants were not known to the interviewers prior to their participation in the study.

A standardized, semi-structured interview guide was developed based on the information obtained from a targeted literature search, blogs/forums covering individual perspectives of HCC, and discussions with clinicians (Online Resource 1). The targeted literature search was conducted using PubMed in October 2018 to identify published research describing the experiences of individuals with HCC. Five patient blog entries/forums detailing symptoms and impacts of HCC (PatientsLikeMe, Blog for a Cure, American Cancer Society, Hepatitis B Foundation, and HealthBoards cancer forums [24–28]) were identified as supplementary sources of information via a targeted search. Telephone discussions were conducted with oncologists from the USA (*n* = 2), Europe (*n* = 2), and Asia (*n* = 1). The clinicians worked in university hospitals or academic centers and had at least 7 years’ relevant practice experience.

During the qualitative interviews, interviewers asked open-ended questions to give participants the opportunity to talk freely about their HCC, including its signs/symptoms and impacts on their daily lives, and asked prompted questions to explore participants’ experiences in greater depth. To examine whether a sign/symptom might be disease and/or treatment related, interviewers probed participants on whether they or their treating physician attributed it to HCC and/or to its treatment, and whether it was experienced before, during, or after treatment. For signs/symptoms and impacts reported by participants, interviewers asked participants how disturbing the sign/symptom or impact was or is to their life using a 0–10 scale, with 0 being ‘not at all disturbing’ and 10 being ‘very disturbing’. When time permitted, participants were also asked to review questions from identified PRO instruments, but this was deprioritized when the concept elicitation part of the interview was lengthy. Interviews lasted approximately 75 to 90 min each and were audio-recorded with the participants’ permission. The study team used de-identified field notes to ensure that there would be no loss of quantitative findings for the analysis. Verbatim transcription of the audio files and general quality control were performed by a professional third party.

### Data analysis

A preliminary codebook for analysis of the qualitative HCC interview transcripts was designed based on the literature searches, blogs/forums review, and discussions with clinicians. The codebook was continuously reviewed and updated as new concepts emerged from the HCC interview transcripts. Two researchers assigned codes to sections of text from the interview transcripts relating to signs/symptoms and impacts of HCC, to identify common themes, using ATLAS.ti software (version 8; ATLAS.ti Scientific Software Development GmbH, Berlin, Germany). Six interview transcripts (from interview numbers 5, 10, 15, 16, 20, and 25) were coded by both researchers, to assess inter-coder agreement and as a basis on which to discuss updates to the codebook and coding rules. Good inter-coder agreement was predefined as Krippendorff’s coefficient alpha binary > 0.7 [29].

Concept analysis was undertaken both in the interviewed group overall and in disease-stage subgroups (BCLC stages A, B, and C). A concept was deemed ‘salient’ if at least 50% of participants mentioned the concept and it had a mean disturbance rating of five or higher (on the 0–10 scale).

The adequacy of the patient sample size was estimated using the principle of concept saturation [20]. Qualitative study data indicate that a minimum sample size of 12–15 is usually sufficient for concept elicitation interviews [30, 31] and having a sample size of more than 25 is generally not considered beneficial [32]. To assess concept saturation, transcripts were grouped chronologically into five waves of five interviews each, and concepts derived from each wave of interviews were compared with concepts from the prior wave(s). If no new concepts appeared in the final wave of interviews, saturation of concepts was considered achieved.

### Conceptual model

Signs/symptoms or impacts related to HCC and/or its treatment were used to construct conceptual models to capture concepts overall and by disease stage. Initial models were created from the reviews of the literature and patient blogs/forums and were refined based on the qualitative interviews. The conceptual models included information on salient concepts overall and by disease stage. Signs/symptoms or impacts that had been deemed by interviewed individuals to be exclusively treatment related were not included in the HCC conceptual model. The reason for excluding exclusively treatment-related concepts was that different patients will experience different treatment regimens, and the objective of this research was to capture the common experience of HCC.

### PRO instrument mapping

The PubMed, ClinicalTrials.gov, PROQOLID, and PROLABELS databases were searched in October 2018 to identify PRO instruments used in HCC studies. PubMed was used to identify development and validation studies in HCC for each identified PRO instrument, to assess their content validity and psychometric properties. Salient signs/symptoms identified during the interviews were mapped to selected PRO instruments to determine how well these instruments captured the salient concepts.

## Results

### Patient interviews

A total of 25 individuals with HCC were interviewed (Table 1). The median age was 63 years (range: 44–79 years), 56% of participants were white, and about two thirds (68%) were men. Three individuals (12%) had BCLC stage A HCC, eight (32%) had stage B HCC, and 14 (56%) had stage C HCC. About two thirds of participants (68%) had received procedures for their HCC, and 88% of participants had previously received or were currently receiving pharmaceutical treatments for their HCC (Table 2).

**Table 1:** Demographic and clinical characteristics of qualitative interview participants

Demographic characteristic	Participants (*N* = 25)
Sex, *n* (%)	
Men	17 (68)
Age, years	
Mean (SD)	63 (8.2)
Ethnicity, *n* (%)	
Black or African American	6 (24)
White or Caucasian	14 (56)
Hispanic	2 (8)
Asian	3 (12)
Region	
Northeast	1 (4)
Midwest	5 (20)
West	7 (28)
South	12 (48)
Education, *n* (%)	
Bachelor/graduate degree	11 (44)
Some college	7 (28)
High school	4 (16)
No high school	1 (4)
Unknown	2 (8)
Work status, *n* (%)	
Employed	3 (12)
Unemployed	8 (32)
Retired	14 (56)
Tumor stage at time of interview	
BCLC stage A	3 (12)
BCLC stage B	8 (32)
BCLC stage C	14 (56)

*BCLC* Barcelona clinic liver cancer, *SD* standard deviation

**Table 2:** Procedures and pharmaceutical therapies received as part of HCC treatment by qualitative interview participants, overall, and by BCLC disease stage

Clinical characteristic	All participants (*N* = 25) n (%)	Stage A (*n* = 3) n (%)	Stage B (*n* = 8) n (%)	Stage C (*n* = 14) n (%)
Procedures received				
TACE/TAE	8 (32)	1 (33)	3 (38)	4 (29)
Radiofrequency ablation	7 (28)	0 (0)	1 (13)	6 (43)
Radioembolization (Y90)	4 (16)	1 (33)	2 (25)	1 (7)
Radiation	2 (8)	0 (0)	1 (13)	1 (7)
Local ablation	1 (4)	1 (33)	0 (0)	0 (0)
Hepatic resection	1 (4)	1 (33)	0 (0)	0 (0)
Liver transplantation	1 (4)	1 (33)	0 (0)	0 (0)
None/unknown	8 (32)	1 (33)	3 (38)	4 (29)
Pharmaceutical treatments received previously				
Cisplatin	2 (8)	0 (0)	0 (0)	2 (14)
Doxorubicin	2 (8)	0 (0)	0 (0)	2 (14)
Nivolumab	4 (16)	0 (0)	0 (0)	4 (29)
Pembrolizumab	1 (4)	0 (0)	0 (0)	1 (7)
Regorafenib	2 (8)	0 (0)	0 (0)	2 (14)
Sorafenib	13 (52)	1 (33)	3 (38)	9 (64)
None/unknown	3 (12)	0 (0)	3 (38)	0 (0)
Pharmaceutical treatments receiving currently				
Cisplatin	1 (4)	0 (0)	0 (0)	1 (7)
Doxorubicin	1 (4)	0 (0)	0 (0)	1 (7)
Durvalumab	1 (4)	0 (0)	0 (0)	1 (7)
Galunisertib	1 (4)	0 (0)	0 (0)	1 (7)
Lenvatinib	1 (4)	0 (0)	0 (0)	1 (7)
Nivolumab	11 (44)	1 (33)	3 (38)	7 (50)
Sorafenib	2 (8)	0 (0)	1 (13)	1 (7)
None/unknown	3 (12)	0 (0)	3 (38)	0 (0)

Participants may have had more than one procedure and/or treatment

*HCC* hepatocellular carcinoma, *TACE* transarterial chemoembolization, *TAE* transarterial embolization, *Y90* radioactive isotope yttrium

Good inter-coder agreement was reached for all but one of the double-coded interview transcripts (transcript 15). A coding resolution meeting was held to discuss discrepancies in preparation for the next transcript to be double coded, and good agreement was subsequently obtained. A total of 73 concepts were identified, of which 67 concepts (92%) arose in the first two waves of interviews. Concept saturation was reached by the fifth and third of five interview waves for signs/symptoms and impacts, respectively.

**Fig. 1:**
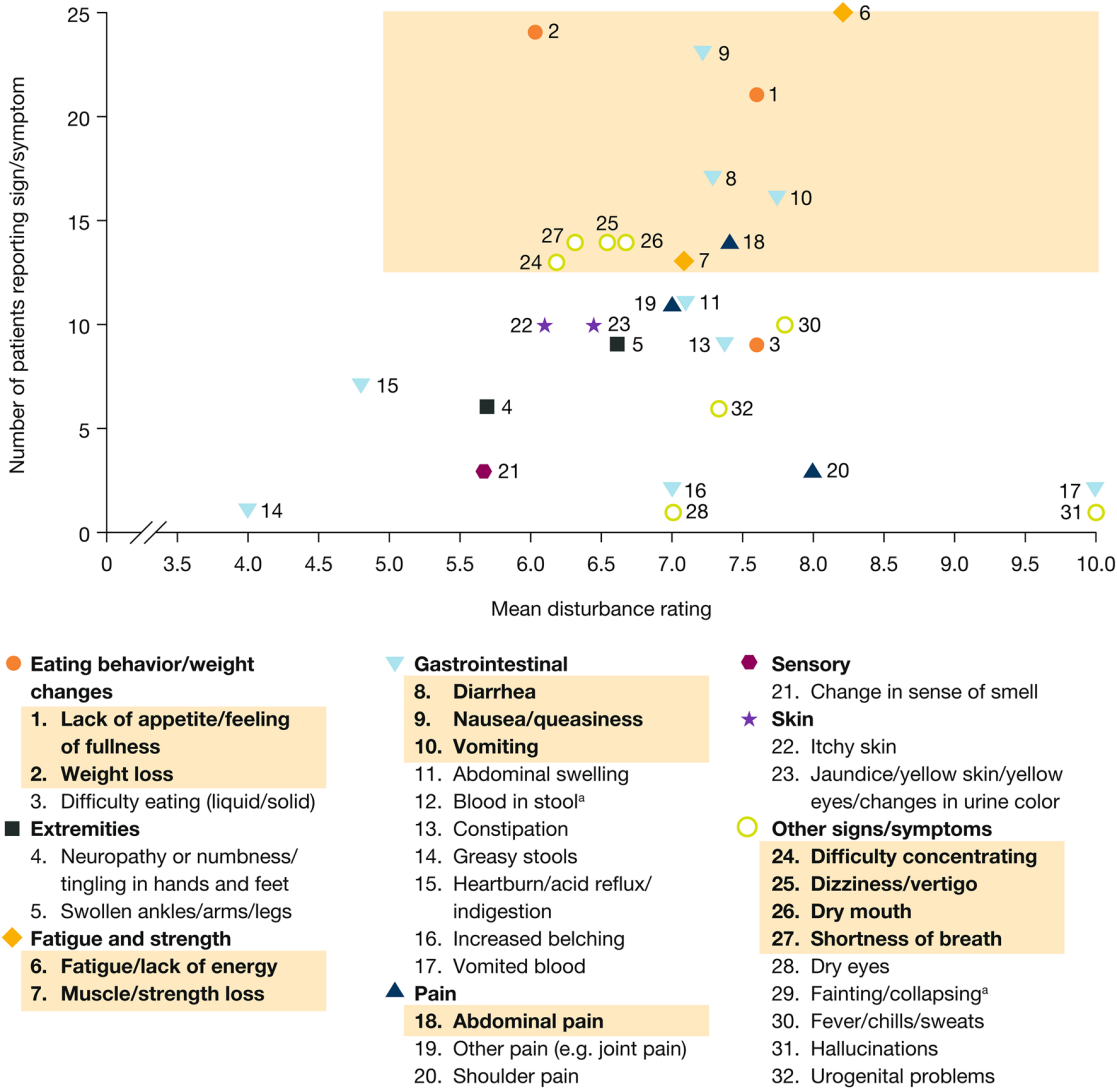
Signs/symptoms related to HCC or to both HCC and its treatment. Shaded area represents salient signs/symptoms. *HCC* hepatocellular carcinoma. ^a^Not shown/not rated

**Fig. 2:**
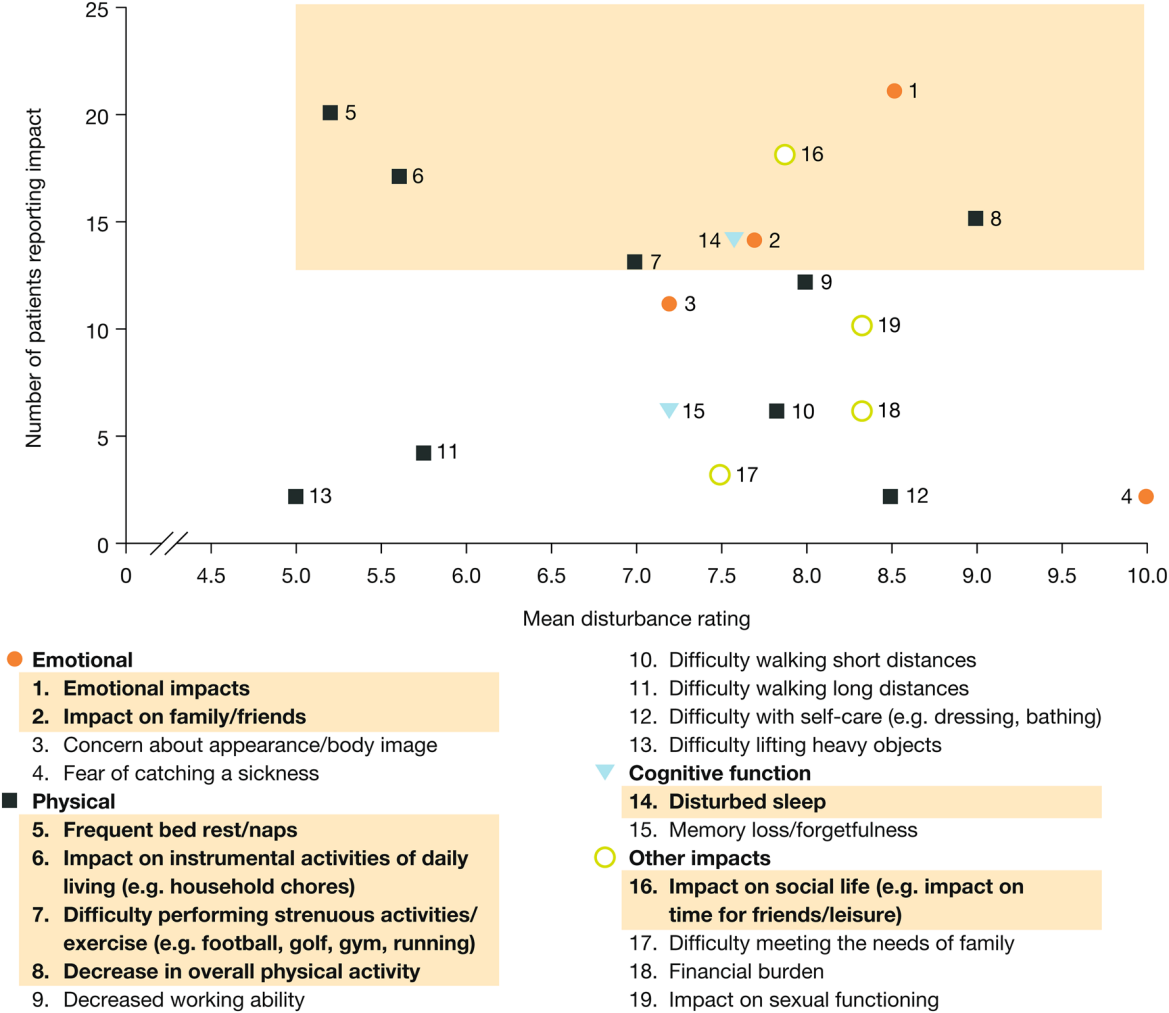
Impacts related to HCC or to both HCC and its treatment. Shaded area represents salient impacts. *HCC* hepatocellular carcinoma

The sign/symptom and impact concepts mentioned are depicted in Figs. 1 and 2, respectively, plotted by the number of individuals mentioning each concept and its mean disturbance rating. Sign/symptoms and impact concepts that reached salience are listed in Tables 3 and 4, respectively, together with the number of individuals mentioning each concept spontaneously or when prompted, the mean disturbance rating, and example quotations. All participants spoke about their fatigue/lack of energy (mean disturbance rating: 8.2), and most (21/25 participants) did so without prompting. Interview participants mentioned needing to take naps during the daytime and not having the energy that they used to have. Almost all individuals (24/25) reported having lost weight (mean disturbance rating: 6.0), with most (21/24) mentioning it spontaneously. For participants who mentioned the amount of weight lost, this ranged from approximately 5 kg to 10 kg, although an individual with stage C disease who had severe diarrhea described losing about 55 kg. Lack of appetite/feeling of fullness (mentioned by 21 participants [19 spontaneously]; mean disturbance rating: 7.6) was sometimes described in conjunction with weight loss and nausea; and nausea/queasiness (mentioned by 23 participants [19 spontaneously]; mean disturbance rating: 7.2) was described as often being accompanied by vomiting. Participants rated abdominal pain as the most disturbing sign/symptom (mentioned by 14 participants [11 spontaneously]; mean disturbance rating: 9.0). Most participants described the emotional impacts of their HCC (mentioned by 21 participants [13 spontaneously]; mean disturbance rating: 8.5). Participants talked about feeling frustrated, depressed, and scared about their disease. In an assessment of elicited concepts by gender, it appeared that women provided spontaneous information on emotional impacts more often than men (results not shown).

**Table 3:** Salient sign and symptom concepts and example quotations elicited from individuals with HCC (*N* = 25)

Sign/symptom	Individuals mentioning sign/symptom, *n* (%)				Mean disturbance rating (number of patients rated)^a^	Example quotation (BCLC stage; participant ID number)
	S	P		Total		
Lack of appetite/feeling full	19 (76)	2 (8)		21 (84)	7.6 (20)	“The biggest one is my appetite. Going from wanting to eat to not is a big difference.” (Stage B; HCC21)
Weight loss	21 (84)	3 (12)		24 (96)	6.0 (24)	“I had the weight loss from naturally not eating as well.” (Stage A; HCC03)
Fatigue/lack of energy	21 (84)	4 (16)		25 (100)	8.2 (24)	“Nothing but just like dog tired. I just thought it was normal. […] Just tired, exhausted. […] No energy.” (Stage C; HCC09)
Muscle/strength loss	5 (20)	8 (32)		13 (52)	7.1 (12)	“I lost everything I had. I’ve got no muscle. I'm skin and bones.” (Stage C; HCC11)
Diarrhea	10 (40)	7 (28)		17 (68)	7.3 (17)	“It's not every day, but with the diarrhea it’s just every now and then but it's quite a bit when I do.” (Stage A; HCC08)
Nausea/queasiness	19 (76)	4 (16)		23 (92)	7.2 (23)	“You feel like you’re going to have dry heaves or something. Your stomach hurts a little bit.” (Stage B; HCC02)
Vomiting	14 (56)	2 (8)		16 (64)	7.8 (16)	“[…] you feel like throwing up and stuff. Sometimes I do. […] It just comes and goes out of nowhere.” (Stage C; HCC11)
Abdominal pain	11 (44)	3 (12)		14 (56)	9.0 (12)	“Like a sharp pain. […] Right on the side, actually. It was right on the side of my stomach. […] The right side.” (Stage C; HCC16)
Difficulty concentrating	2 (8)		11 (44)	13 (52)	6.2 (11)	“[…] because I’d just be wondering what’s going to happen to me. I can’t concentrate. My mind wanders constantly.” (Stage C; HCC09)
Dizziness/vertigo	3 (12)		11 (44)	14 (56)	6.4 (13)	“I don’t know, but sometimes I’m on the couch watching TV, and then I try to get up to go to the bathroom and then boom. It hits me, the dizziness.” (Stage C; HCC16)
Dry mouth	2 (8)		12 (48)	14 (56)	6.7 (12)	“It’s constant thirst and dry mouth.” (Stage C; HCC18)
Shortness of breath	5 (20)		9 (36)	14 (56)	6.3 (12)	“But, yeah, I did have […] mainly because of the fatigue and lack of stamina, I had some shortness of breath.” (Stage B; HCC02)

^a^For some interviews, insufficient time was available to discuss all disturbance ratings. Consequently, the number of individuals who mentioned a disturbance rating for a sign/symptom does not always equal the number of individuals who mentioned the sign/symptom

*BCLC* Barcelona clinic liver cancer, *HCC* hepatocellular carcinoma, *P* prompted, *S* spontaneous

**Table 4:** Salient impact concepts and example quotations elicited from individuals with HCC (*N* = 25)

Impact	Individuals mentioning impact *n* (%)			Mean disturbance rating (number of patients rated)^a^	Example quotation (BCLC stage; participant ID number)
	S	P	Total		
Disturbed sleep	2 (8)	12 (48)	14 (56)	7.6 (12)	“Emotionally, it affects me sleeping-wise. I have nightmares a lot of the time, so it does affect me.” (Stage C; HCC17)
Emotional impacts	13 (52)	8 (32)	21 (84)	8.5 (19)	“But probably the psychological aspect of the cancer has affected me more than anything else. That’s my identity now, is being a cancer patient versus being who I was before. […] The psychology of cancer is worse than the cancer itself in many ways.” (Stage B; HCC02)
Impact on family/friends	11 (44)	3 (12)	14 (56)	7.7 (10)	“I’d rate it a 10 because he has to take care of me. And I never thought that would happen.” (Stage A; HCC08)
Impact on social life (e.g., impact on time for friends/leisure)	13 (52)	5 (20)	18 (72)	7.9 (16)	“With the cancer and with the diarrhea and with the things the cancer brings on to your body, it’s just you’re afraid to go out to a club or to a restaurant.” (Stage B; HCC10)
Frequent bed rest/naps	8 (32)	12 (48)	20 (80)	5.2 (19)	“I try to get eight hours [sleep each night]. Sometimes I take naps even when I go in to work, I’ll go out to my truck and take a nap.” (Stage C; HCC20)
Impact on instrumental activities of daily living (e.g., household chores)	6 (24)	11 (44)	17 (68)	5.6 (13)	“Before, I used to vacuum my house every other day. Now I just stick to doing it Sundays.” (Stage B; HCC25)
Difficulty performing strenuous activities/exercise (e.g., football, golf, gym, running)	7 (28)	6 (24)	13 (52)	7.0 (10)	“My exercise is zero, just no energy.” (Stage C; HCC058)
Decrease in overall physical activity	11 (44)	4 (16)	15 (60)	9.0 (2)	“Say I could walk to check the mailbox. I could come in and go out. Now I will go check the mailbox and I’ll kind of stand out there for a couple minutes and then I’ll come back inside.” (Stage B; HCC25)

^a^For some interviews, insufficient time was available to discuss all disturbance ratings. Consequently, the number of individuals who mentioned a disturbance rating for an impact does not always equal the number of individuals who mentioned the impact

*BCLC* Barcelona clinic liver cancer, *HCC* hepatocellular carcinoma, *P* prompted, *S* spontaneous

### Conceptual model of the HCC patient experience

**Fig. 3:**
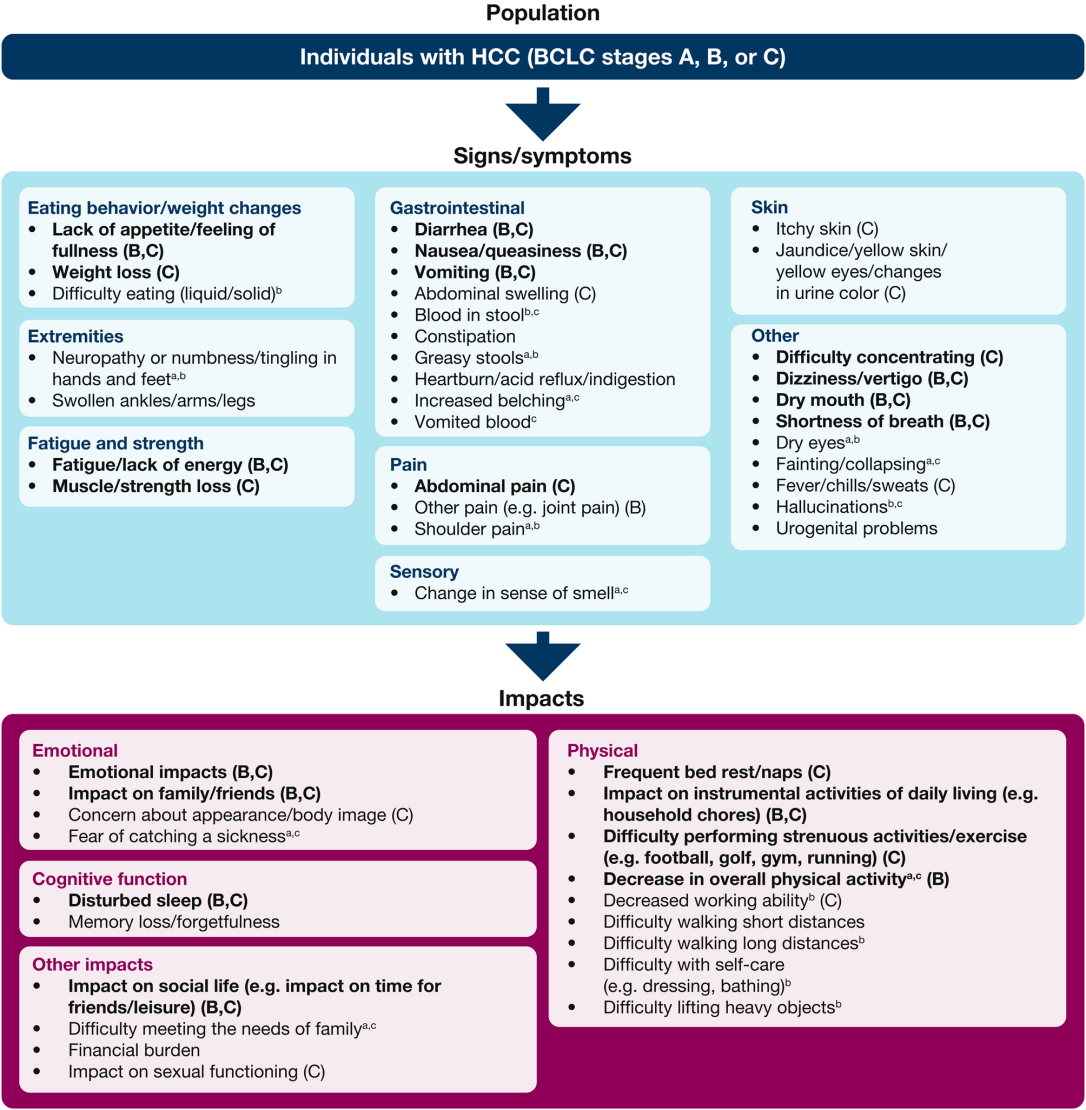
Disease-specific conceptual model for HCC, BCLC stages A–C. Concepts shown in bold font are those that were salient overall. Concepts followed by bracketed B and/or C are those that were salient in BCLC stages B and/or C, respectively. No conclusions about salience were drawn for stage A because only three individuals had stage A disease. *BCLC* Barcelona clinic liver cancer, *HCC* hepatocellular carcinoma. ^a^Concept was not mentioned by individuals with stage A disease. ^b^Concept was not mentioned by individuals with stage B disease. ^c^Concept was not mentioned by individuals with stage C disease

The all-stage conceptual model for HCC comprises 32 sign/symptom concepts and 19 impact concepts related to HCC or to both HCC and its treatment, organized into 12 categories: eating behavior/weight changes (3 concepts), extremities (2 concepts), fatigue, and strength (2 concepts), gastrointestinal (10 concepts), pain (3 concepts), sensory (1 concept), skin (2 concepts), other symptoms (9 concepts), emotional (4 concepts), physical (9 concepts), cognitive function (2 concepts), and other impacts (4 concepts) (Fig. 3).

Twenty-one signs/symptoms (66%) and 12 impacts (63%) were elicited by interview participants in all three disease stages (Fig. 3). Twenty concepts were salient overall, comprising 12 signs/symptoms (lack of appetite/feeling of fullness, weight loss, fatigue/lack of energy, muscle/strength loss, diarrhea, nausea/queasiness, vomiting, abdominal pain, difficulty concentrating, dizziness/vertigo, dry mouth, shortness of breath) and eight impacts (emotional impacts, impact on family/friends, frequent bed rest/naps, impact on instrumental activities of daily living, difficulty performing strenuous activities/exercise, decrease in overall physical activity, disturbed sleep, and impact on social life) (Fig. 3).

Although the participant numbers in the disease stage subgroups were small, the interview data suggested some differences in experiences across various stages of HCC (Fig. 3). For example, weight loss, muscle strength loss, and frequent bed rest/naps were salient in stage C but not in stage B; decrease in overall physical activity was salient only in stage B; and abdominal pain, difficulty concentrating, and difficulty performing strenuous activities/exercise were salient only in stage C. Several concepts were salient in one or two disease stages but did not reach overall salience. For example, salience in stage B, but not overall, was obtained for other pain (e.g., joint pain). Salience in stage C, but not overall, was observed for skin-related signs/symptoms (itchy skin, jaundice/yellow skin/yellow eyes/changes in urine color), fever/chills/sweats, concern about appearance/body image, and decreased working ability.

### PRO instrument mapping

The EORTC QLQ-C30 [12], together with its HCC-specific module QLQ-HCC18 [13], and the Functional Assessment of Cancer Therapy-General (FACT-G) [33], together with its hepatobiliary cancer-specific module FACT-Hep [34], were identified as the most commonly used disease-specific PRO instruments in HCC studies. The decision was made to focus on one set of questionnaires, in order to keep interview times to a manageable length. The EORTC QLQ-C30 plus QLQ-HCC18 together were prioritized over the FACT-G plus FACT-Hep for the current study because of slightly greater coverage of relevant concepts identified from the literature review.

Nine of the interviewed individuals with HCC were asked to review questions from the Physical Functioning and Fatigue domains of the EORTC QLQ-C30 and QLQ-HCC18. All nine individuals reported that all questions were easy to understand and relevant to their HCC experience, despite not all of them reporting that they had experienced all concepts in the domains within the instruments’ 1-week recall period.

Mapping of the 12 salient signs/symptoms to the EORTC QLQ-C30 and QLQ-HCC18 showed that 10 of these were covered by these PRO instruments. The only two salient signs/symptoms that were not covered were dizziness/vertigo and dry mouth.

## Discussion

Engaging people living with HCC is crucial to understanding the disease experience fully and identifying signs/symptoms and impacts that are relevant to the patient experience. This qualitative study included in-depth interviews with individuals with HCC to obtain a rich understanding of the patient experience of HCC and to guide fit-for-purpose patient-centered HCC outcome measurement in clinical development. A conceptual model of HCC was developed to describe the signs/symptoms and impacts of HCC experienced by patients, overall and by disease stage. To our knowledge, this is the first conceptual model for HCC that also provides insights per disease stage, from early stage (BCLC stage A) to advanced stage (BCLC stage C) HCC.

Of the 51 signs/symptoms and impacts in the HCC conceptual model, 20 concepts were considered salient, that is, they were prevalent and highly disturbing. However, although these concepts were considered salient based on the interviews conducted with people with HCC, individual experiences may vary and this should be accounted for when developing PRO measurement strategies for clinical trials, as determined by specific context of use. Although the presence or history of cirrhosis was not captured in the present study, a significant proportion of patients diagnosed with HCC have pre-existing liver disease and cirrhosis in addition to their tumor burden [35]. Some of the reported signs/symptoms and impacts may, thus, have been caused or amplified by underlying liver disease and their presence and magnitude may, therefore, differ in patients with HCC depending on whether cirrhosis is present or absent.

Research across cancer types has shown that fatigue is one of the most frequent and distressing symptoms of cancer and its treatment [36, 37]. Consistent with these findings, in the current study, fatigue/lack of energy was identified across the various disease stages as being a particularly prevalent experience that individuals with HCC found highly disturbing. Findings per disease stage illustrated that abdominal pain and skin-related issues, which were rated as highly disturbing, were particularly prevalent in individuals with stage C disease, most likely reflecting the increasing tumor burden and liver damage in this disease stage. The most prevalent and disturbing impacts of HCC across the various disease stages were the emotional impacts of the disease, including frustration, fear, and depression, as well as impacts on social life, family and friends, and important activities of daily living. In support of current study findings, the Taiwanese study of 33 individuals treated for HCC participants described feelings of anger, anxiety and depressive mood, and disrupted and strained social and family relationships [10]. Although the current study did not include individuals with stage D HCC, findings are consistent with interviews conducted with people with terminal stage HCC who described their disease experience as all-consuming and talked about feeling hopeless, annoyed and isolated [38]. In accordance with the Taiwanese study, in which participants described a lack of physical vigor [10], physical impacts were frequent in the current study: findings per disease stage showed that a decrease in overall physical activity was common and disturbing for individuals with stage B HCC, and difficulty performing strenuous activities or exercise and decreased working ability were common and disturbing for those with stage C disease.

Women appeared to provide unsolicited information on emotional impacts more often than men, indicating that asking about these impacts is relevant and should be made a routine part of practice.

PRO measures are needed to capture the signs/symptoms and impacts of a disease or condition directly from patients. The EORTC QLQ-C30 and its HCC-specific module QLQ-HCC18, and the FACT-G/FACT-Hep [34] were identified as the most commonly used disease-specific PRO instruments in HCC studies. The EORTC QLQ-C30/QLQ-HCC18 were shown in the current study to capture the relevant signs/symptoms that are most meaningful to patients with HCC, and to be fit for purpose. Of the 12 salient signs/symptoms elicited from individuals with HCC, 10 are covered by these EORTC instruments. The FACT-G and its hepatobiliary cancer-specific module FACT-Hep were not further evaluated in the current study. While there is commonality between the EORTC QLQ-C30/QLQ-HCC18 and the FACT-G/FACT-Hep in terms of conceptual coverage, there are some gaps in both forms of questionnaires, such as that the FACT-G/FACT-Hep do not cover muscle/strength loss, vomiting, difficulty concentrating or shortness of breath, the EORTC QLQ-C30/QLQ-HCC 18 do not cover dry mouth, and none cover dizziness/vertigo. Researchers should consider disease- and treatment-related criteria, including those of novel targeted therapies, within the context of use when deciding on PRO questionnaire strategy. Additional PRO items should be considered as supplementary tools to evaluate fully the impact of the disease and treatment on patients’ experience given a specific context of use [39–41]. Supplemental items to capture the patient experience can be obtained from the EORTC quality of life item library, the Patient-Reported Outcome Measurement Information System (PROMIS) cancer item bank, and the PRO-Common Terminology Criteria for Adverse Events (PRO-CTCAE) item library.

This study has several important strengths. It had a specific focus on HCC. Rigorous qualitative methodologies were used in the qualitative interviews. Concept saturation was reached, indicating an adequate sample size for the qualitative interviews. Although qualitative study data indicate that a minimum sample size of 12–15 is usually sufficient for concept elicitation [30, 31], a limitation of this study is the small number of participants specifically in the early disease setting (BCLC stage A; *n* = 3). The study did not include individuals with very early stage (stage 0) or terminal stage (stage D) HCC. While significant efforts were made to obtain representation across the HCC spectrum, recruitment proved challenging despite partnering with a patient advocacy organization. This was particularly the case for individuals with early stage HCC, as HCC is often diagnosed only at advanced disease stage. All participants were from the USA and were, thus, not representative of the geography of the disease, which is more common in Asia and Africa than in other parts of the world. Despite being fairly representative of US demographics, the sample was skewed toward white participants, which is a demographic limitation from the perspective of global demographics. Participants were asked about whether they attributed a concept to HCC and/or to its treatment and, although exclusively treatment-related concepts were excluded from the HCC conceptual model, it is likely that the treatment regimens experienced by different individuals also affected the signs/symptoms and impacts of HCC that were reported.

In conclusion, individuals with HCC reported a range of signs/symptoms and impacts, such as emotional and physical distress that negatively affect their daily functioning and quality of life. Obtaining rich qualitative patient experience data directly from people living with the disease guides patient-centered outcome measurement and can help inform patients about what to expect during treatment. The results provide healthcare professionals with important information to contribute to collaborative treatment decision making and patient education. Including patient-reported outcome measures such as the EORTC QLQ-C30 and QLQ-HCC18 in HCC drug development has the potential to provide meaningful patient perspectives, complementing traditional efficacy and safety outcome measures in clinical trials. In light of recent advancements in cancer therapeutics, such as targeted therapies, researchers and healthcare professionals may wish to ensure that relevant treatment-related symptoms are appropriately evaluated.

## Supplementary Information

Below is the link to the electronic supplementary material.

Supplementary file1 (PDF 362 kb)

## Data Availability

The data supporting the findings are available within the article.
