# Supplementary material for: Understanding the patient experience in hepatocellular carcinoma: a qualitative patient interview study
Source: Qual Life Res. 2021 Jun 11;31(2):473–85. doi: 10.1007/s11136-021-02903-4 (PMC8847294; doi:10.1007/s11136-021-02903-4)
Supplement: Supplementary file 1 — Supplementary file1 (PDF 362 kb) [file 11136_2021_2903_MOESM1_ESM.pdf]

IMS Health & Quintiles are now IQVIA™

PATIENT INTERVIEW DISCUSSION GUIDE FOR PATIENTS WITH HEPATOCELLULAR CARCINOMA (HCC)

**TITLE**	Interview Guide: Qualitative interviews with patients who suffer from hepatocellular carcinoma (HCC)
**VERSION**	13 December 2018
**CONDUCTED BY**	IQVIA Consulting Services<br>300 Vesey Street, 13th Floor<br>New York, New York 10282
**RESEARCH TARGET**	30 or more patients until concept saturation is reached in the US

IMS Health & Quintiles are now IQVIA

[For internal purposes only] *Discussion overview and objectives*

Hepatocellular Carcinoma Patient Interviews (75 - 90 minutes)			
Guide Section	***Objectives***	***Output Expectation***	***Timing***
1. Introduction	• Establish objective & expectations for discussion	• Consent for discussion and recording	*2 min*
2. Participant Background	• Determine patient demographic information	• Patient demographics	*3 min*
3. Disease Background	• Determine circumstances and timing of initial diagnosis<br>• Determine current disease stage and disease history<br>• Verify current and all prior treatments	• Patient disease history (i.e. initial signs / symptoms, prior treatment / intervention, disease recurrences)	*10 min*
4. Signs, Symptoms, and Impacts	• Gather spontaneously reported signs, symptoms and impacts<br>• Probe on signs, symptoms, and impact from literature review and / or prior interviews not stated by patient<br>• Ascertain disturbance level on patient's quality of life<br>• Probe on current concepts and also past concepts relating to key events in disease history if patient can recall	• Spontaneous signs, symptoms, and impacts<br>• Prompted signs, symptoms, and impacts<br>• Disturbance rating<br>• Symptoms and impacts across disease stages	*50 min*
5. PRO Questionnaires and meaningful change	For fatigue, physical functioning and sleep problems/ insomnia:<br>• Administer relevant questions from the EORTC QLQ-C30, HCC18 and PROMIS questionnaire to participants<br>• Explore the relevance of the questions and the scales to the patient's experience along their disease journey<br>• Discuss the patients' high-level perspective on meaningful change for each item alone and as part of the scale	For fatigue, physical functioning and sleep problems/ insomnia:<br>• Patient assessment of content validity of selected items in the questionnaires<br>• Qualitative insights of what constitutes meaningful change on the items and concepts	*20min*
6. Close	• Thank and close		*5 min*

Key: ***Moderator Note*** **/** ***Moderator Script*** **/** [Key Topic to Test]

0. Document Purpose

Moderator Note: This document is intended to be used to guide semi-structured interviews with patients to explore the signs, symptoms, and impacts of hepatocellular carcinoma (HCC) at different stages of the disease. Interviews will be performed individually over the phone and are anticipated to take approximately 75 – 90 minutes. Interviews will be conducted in English by experienced IQVIA researchers. Interviews will be audio recorded to enable transcripts development.

1. Interview Introduction (2 minutes)

Moderator Scripts:

Hello,

This is [Interviewer Name] from IQVIA. I am reaching out to discuss your experience with liver cancer. This interview will take 75-90 minutes. Is this still a good time to talk?

[If no] – Is there another time that works for you? [Reschedule as appropriate]

[If yes – Proceed below]

Before we begin, I want to re-confirm that it is okay for us to record the interview to generate an interview transcript. May I audio record the interview?

[Pause]

[If no] – Thank you for your willingness to participate however because interview transcripts are required for our study we will not be able to proceed [Terminate interview]

[If yes – proceed below]

*I'd also like to remind you only to **use first names** when we begin recording if you refer to friends or family in any of your responses. This will help keep the interview anonymous. In addition to recording, we may have a few listeners from our project team on what is called a listen-only line. While they will not be active participants in our conversation, they are very interested in what you have to say. Is it okay for other project team members to listen to the call? [Pause]*

[If yes: Proceed with listen-only line]

[If no: Request IQVIA members to leave the call and proceed below]

Today, we will be discussing your experiences with primary liver cancer, also known as hepatocellular carcinoma (HCC). Is there a particular term you prefer – liver cancer, hepatocellular carcinoma or HCC or something else? [Interviewer to use the term that patient is most comfortable, to replace liver cancer with throughout discussion]. The output from this interview may contribute to the development of new liver cancer [or term used by patients] treatments.

This interview has four main sections:

1. *First, we will talk about your general experience with your condition, including diagnosis, treatment history, and current symptoms*
2. *Next, we will talk in more depth about your symptoms and how they have impacted your life*
3. *Third, you will be asked to complete a couple of questionnaires that are being considered for use in research studies and clinical trials with individuals with your condition*
4. *Finally, after you have completed the questionnaires, I will ask you whether the questions are relevant to how you experience your condition and what level of improvement on these questions would be meaningful to you*

Before we begin, I want to ensure you have access to the screen sharing platform that will be used at the end of the session for you to complete the questionnaires.

Moderator Note: *Please ensure participant has access to the relevant sharing platform [join.me or appropriate ClearSlide] before starting the interview*

If at any time during this interview I ask you a question that you do not want to answer or makes you feel uncomfortable, please let me know and we can switch subjects or end the interview. Do you have any questions before we begin? [Pause for questions]

Moderator Note: Start Recording

2. Participant Background (3 minutes)

Moderator Script*: Before we discuss your experience with liver cancer [or term used by patient], I would like to ask you a few questions about yourself.*

2.1. Can you please tell me your **first name** and **your last initial** only?

2.2. What is your Age?

2.3. Gender?

2.4. Race?

2.5. State of residence?

2.6. Education level?

2.7. Marital status?

3. **Disease Background (10 minutes)**

3.1. How do you describe your disease when you discuss it with others?

3.2. How long have you known about your liver cancer [or term used by patient] (i.e. when were you first diagnosed)?

3.3. What initially brought you to the doctor when you were first diagnosed?

PROBE:

- If due to a routine surveillance
- If in response to symptoms*. [If symptoms, record on disturbance form as spontaneous but to not probe at this point]*

3.4. Since your diagnosis, has your doctor indicated that your liver cancer [or term used by patient] has gotten worse? Could you take us through your disease progression journey?

PROBE: *[If disease progressed]*

- How did your disease progress or change over time?
- What led to the determination of disease progression? *[If symptoms, record on disturbance form as spontaneous but to not probe at this point]*

3.5. Since your diagnosis, has your doctor indicated that your liver cancer [or term used by patient] has spread to other areas of your body?

PROBE:

- When were you first told that your liver cancer [or term used by patient] spread?
- What part(s) of the body has it spread to?
- Were there any symptoms associated with its spread? *[If symptoms, record on disturbance form as spontaneous but to not probe at this point]*

3.6. What treatments or procedure have you received to treat your liver cancer [or term used by patient]?

3.6.1. What procedure / treatment did you receive after you were diagnosed with liver cancer [or term used by patient]?

3.6.2. What other procedures / treatments have you received?

3.6.3. What treatments are you currently receiving?

3.6.4. How long have you been receiving these treatments?

3.6.5. Why did you / your doctor determine that you needed this procedure / treatment *[If symptoms, record on disturbance form as spontaneous but to not probe at this point]*

PROBE:

- Chemotherapy
- Targeted drug therapies (e.g., sorafenib / Nexavar®, lenvatinib / Lenvima®, regorafenib / Stivarga®, nivolumab / Opdivo ®)
- Drugs to help cope with side-effects from other therapies
- Surgical procedures (e.g., hepatic resection, radiofrequency ablation, transplant / local ablation)
- Transcatheter arterial chemoembolization (TACE)

3.7. If not mentioned previously, have you experienced a recurrence (i.e. has the cancer come back after a period of time during which the cancer could not be detected?

3.7.1. When did this recurrence occur?

3.7.2. What brought you to the doctor at the time of recurrence?

PROBE:

- If due to a routine surveillance
- If in response to symptoms*. [If symptoms, record on disturbance form as spontaneous but to not probe at this point]*

4. Signs / symptoms / impact (50 minutes)

Moderator Note: *Among adjuvant patients where patients may be symptomatic, please go deeper into impacts as they are more likely to be affected by their condition. This will be informative to prioritize relevant concepts for study in adjuvant setting.*

Moderator Script: *Let's now discuss in greater detail the symptoms you have experienced in the past or are currently experiencing due to your liver cancer [or term used by patient]. We will start the discussion on each symptom that you are currently experiencing (i.e. in the past month), then we will ask you to recall a few times in the past. We will go through each of your symptoms one by one.*

<u>SPONTANEOUS SYMPTOMS</u>

Moderator Note: *Note symptoms discussed in this section as* ***Spontaneous****, and note whether the respondent has identified them as symptoms of the disease. Be prepared to note symptoms offered by the respondent in his / her exact words.*

4.1. ***Moderator note:*** If patient has not mentioned any symptoms yet: What symptoms are you currently experiencing? ***Moderator note:*** *if the patient has already mentioned any symptoms, start discussing these symptoms first instead.*

Moderator Note: *For each symptom, repeat question 4.2 to 4.10 (if applicable)*

4.2. What is it like when you experience the symptom? Describe what it feels like when you experience this symptom. Can you give an example of a time when this occurred recently (i.e. past month)?

4.3. When did the symptom first start? When did you first experience this symptom? Did you experience this symptom other times as well? Tell me about when.

4.4. How often do you experience this symptom?

PROBE:

- Time of occurrence (e.g., morning, afternoon, evening, night, throughout the day / night?
- Duration of the symptom?

4.5. Does the symptom vary over time (e.g. does it change in intensity, or is it constant)?

PROBE:

- Is there anything that triggers or helps ease the symptom?
- What makes the symptom worse?
- What makes the symptom last longer?
- What do you do to help ease this symptom?

4.6. Did the symptom appear suddenly or did it gradually appear?

4.7. In addition to being associated with liver cancer [or term used by patient], do you feel, or has your doctor expressed that this symptom is because of treatments you are taking or have taken to deal with liver cancer [or term used by patient]? and if so which ones?

4.8. Do you think, or has your doctor expressed that this symptom arises from other conditions that you may be suffering from and not because of your liver cancer [or term used by patient]?

4.9. On a scale of 0-10, where 0 means not at all disturbing and 10 means greatly disturbing, how disturbing is this symptom now?

4.10. On a scale of 0-10, where 0 means not at all disturbing and 10 means greatly disturbing, how disturbing was this symptom at its peak?

4.11. Are there any other symptoms you are currently experiencing?

Moderator Note: *For each symptom, repeat question 4.2 to 4.10 (if applicable)*

Moderator Script: *Let's now discuss how your symptoms have changed at key points in your treatment. Please only elaborate on what you can recall clearly. If you cannot remember, that is not a problem.*

Moderator note: *For all symptoms already mentioned, please repeat question 4.12. Move on to the next section if patient cannot recall any historical symptom clearly.*

4.12. Has the symptom improved, worsened, or stayed the same over time? Please elaborate how it changed and what you associated with the change in experience (e.g. start/ end of treatment, recurrence)? ***Moderator Note:*** *Please use this question to identify relevant events in the past to discuss the symptoms. Based on these events, please select the relevant probes*

PROBE:

- Severity/ frequency prior to starting TACE/ targeted therapy
- Severity/ frequency while on TACE/ targeted therapy
- Severity/ frequency prior to the resection/ablation/liver transplant
- Severity/ frequency one week after the resection/ablation/liver transplant
- Severity/ frequency three months after the resection/ablation/liver transplant
- Severity/ frequency prior to your doctor diagnosing progression/ recurrence of the disease
- Severity/ frequency after progression/ recurrence of the disease

4.13. Did you experience any other symptoms in the past that you have not mentioned? ***Moderator Note****: Please select probes that are relevant based on patient's earlier answers, the screener and the confirmation of diagnosis form. For each identified symptom, repeat question 4.2 to 4.10 and 4.12 (if applicable).*

PROBE:

- Symptoms at time of diagnosis
- Treatment related symptoms or side effects associated with TACE, targeted therapy (nivolumab, lenvatinib, etc), resection, ablation, transplant, progression or recurrence

PROMPTED SYMPTOMS

Moderator Note: *If respondent reports experiencing the symptom after prompting, mark symptom as PROBED, and note whether the respondent has identified it as a symptom of the disease.*
Focus first on the prioritized symptoms (the symptoms in bold see the ***Appendix-1*** *at the end to this guide) that patients did not discuss in the unprompted section. For these prioritized symptoms, ask the patients whether they experienced the symptom, and if so, ask all in-depth questions asked for unprompted symptoms. Once all prioritized symptoms covered, move to the non-prioritized symptoms. For these symptoms, first ask whether the patients experienced these symptoms and if so, what disturbance rating they would give it. If the disturbance level is higher than 5 out of 10, follow-up will all the in-depth questions.*

Moderator script*: Now I am going to read a list of other symptoms that patients with liver cancer [or term used by patient] mentioned they experienced in previously conducted research.*

4.14. Did you experience any of the following symptoms? For each symptom I read, please tell me if you experienced it in the past month with a simple yes / no. ***Moderator Note:*** *Read out all prioritised symptoms that have not yet been discussed (bolded symptoms in Appendix-1 at the end of this guide). Repeat question 4.2 to 4.10 and 4.12 (if applicable) for each prioritised symptom in the conceptual model. Then read out all non-prioritised symptoms that have not yet been discussed (nonbolded symptoms in Appendix-1 at the end of this guide). Repeat question 4.10. If the disturbance level is higher than 5 out of 10 or the patient notes the symptom is considered disturbing, repeat question 4.2 to 4.9 and 4.12 (if applicable) follow-up will all the in-depth questions.*

UNPROMPTED IMPACTS

Moderator Script: *We've just discussed the symptoms that you have experienced or are currently experiencing due to liver cancer [or term used by patient]. Now I'd like to ask you in what way(s) your life has been impacted or affected by liver cancer [or term used by patient] and/or the related symptoms. Please note, these are not the symptoms we discussed previously which are directly caused by disease but rather other types of effects that liver cancer [or term used by patient] or its treatment may have on your life.*

Moderator Note: *Note impacts discussed in this section as SPONTANEOUS. Obtain description of each impact offered by the respondent in his / her exact words.*

4.15. How is liver cancer [or term used by patient] currently affecting your life and daily functioning?

Moderator Note: *if patient indicates that they do not understand question, provide following information: [These may relate to activities that you used to be able to perform but can no-longer perform]. If patient still does not understand the question, provide the following information [these impacts on your life may be associated with your work, your leisure activities, your social life, your financial affairs, or your emotional wellbeing].*

Moderator Note*: For each impact, repeat question 4.16 to 4.20*

4.16. How often did *[insert the specific impact]* occur (if applicable)?

4.17. Of all the symptoms we discussed, which symptom do you think is the underlying cause of this impact?

Moderator Note: *If the word "disturb" is unclear to the participant, use the terminology "negatively impact" instead.*

4.18. On a scale of 0 to 10, where 0 means does not disturb your life at all and 10 means greatly disturbs your life, how much does this impact disturb your life now?

4.19. Recall a time when this impact was at its peak, can you share with me when and why was that the case?

4.20. On a scale of 0 to 10, where 0 means does not disturb your life at all and 10 means greatly disturbs your life, how much did this impact disturb your life at its peak?

4.21. Are there any other impacts you are currently experiencing? ***Moderator Note****: For each impact, repeat question 4.16 to 4.20*

Moderator Script: *Let's now discuss how these impacts have changed at key points in your treatment. Please only elaborate on what you can recall clearly. If you cannot remember, that is not a problem.*
Moderator note: *For all symptoms already mentioned, please repeat question 4.22. Move on to the next section if patient cannot recall any historical impact clearly.*

4.22. How has the impact you described changed over time? (e.g., before / after treatment, resection, TACE, recurrence.) ***Moderator Note****: Please go back to the same events that were discussed for symptoms in question 4.12 and explore the impacts at that time*

4.23. Did you experience any other impacts in the past that you have not mentioned? ***Moderator Note****: Please select probes that are relevant based on patient's earlier answers, the screener and the confirmation of diagnosis form. For each identified impact, repeat question 4.16 to 4.20 and 4.22 (if applicable).*

PROBE:

- Impacts at time of diagnosis
- Impacts associated with TACE, targeted therapy, resection, ablation, transplant, progression or recurrence

PROMPTED IMPACTS

Moderator Note: *If respondent reports experiencing the impact after prompting, mark impact as PROBED.*

Moderator script*: Now I'm going to read a list of additional ways, that in previously conducted research, patients with liver cancer [or term used by patient] talked about their lives being affected by symptoms and/or their disease.*

4.24. Did you experience any of the following impacts? For each impact I read, please tell me if you experienced it in the past month with a simple yes / no. ***Moderator Note:*** *Read out all prioritised impacts that have not yet been discussed (the bolded impacts in the* ***Appendix-2*** *at the end to this guide. Repeat question 4.16 to 4.20 for each prioritized impact in the conceptual model that the patient has experienced. Then read out all non-prioritised impacts (the non-bolded impacts in the* ***Appendix-2*** *at the end to this guide) that have not yet been discussed. Repeat question 4.18 for each non-prioritised impact that the patient has experienced.*

5. *PRO Questionnaires Review* (20 minutes)

Moderator script: *Thank you. Let's move to the final part of the interview. Now we will be focusing our discussion on a few questions being considered for use in research studies and clinical trials for adults with liver cancer [or term used by patient]. I will be sharing the questionnaires with you, and I would like you to answer the questions on the questionnaire based on* ***your current experience (i.e. in the past week)****.*

Moderator Note: *Set up selected questionnaire questions focusing on physical functioning, fatigue and sleeping problems/ insomnia. Present questions one by one and allow the patient time to provide his or her answer to the question before moving to the next question (you may need to read the questions aloud and collect the patient answer, if so, please read the question exactly as they appear on the screen and do not provide any additional clarification).*

Moderator script: *I will display the questions from the questionnaires on the screen. Please let me know if you are ever unable to view the questions through the screen share program. Please take as much time as you need, and please feel free to think aloud as you answer each question. After your response, we will then discuss each question in more detail before moving on to the next.*

Moderator Note: *Use the table below and for each item addressing prioritized concepts such as fatigue and physical functioning ask the relevant questions.*

Global physical functioning	○	○ How much difficulty do you have doing your daily physical activities, because of your health?
Physical functioning	○ Do you have trouble doing strenuous activities like carrying a heavy shopping bag or suitcase?<br>○ Do you have trouble taking a long walk?<br>○ Do you have trouble taking a short walk outside the house?<br>○ Do you need to stay in bed or chair during the day?<br>○ Do you need help with eating, dressing, washing yourself or using the toilet?	○ Are you able to climb several flights of stairs?<br>○ Does your health now limit you in doing vigorous activities, such as running, lifting heavy objects, participating in strenuous sports?<br>○ Does your health now limit you in doing heavy work around the house like scrubbing floors, or lifting or moving heavy furniture?<br>○
Fatigue	○ Have you felt weak?<br>○ Did you need to rest?<br>○ Were you tired?	○
Sleeping problems/ insomnia	○ Have you had trouble sleeping?	
Role functioning are home, work, or social settings	○ Were you limited in doing either your work or other daily activities?<br>○ Were you limited in pursuing your hobbies or other leisure time activities?	

Moderator Note: *First ask questions 5.1 to 5.3 for all EORTC QLQ-C30 questions relating to physical functioning, and then also for the PROMIS physical functioning items. Then continue to questions 5.5 to 5.11.Then move on to the concept of fatigue and ask questions 5.1 to 5.3 for all EORTC QLQ-C30*

questions relating to fatigue. Then debrief the domain fatigue through questions 5.12 to 5.16. Then debrief the sleeping problems/ insomnia item through questions 5.1 to 5.3 and question 5.17.

5.1. What response would you choose based on your experience today?

5.2. What made you choose that response?

5.3. How relevant is this question to your experience with liver cancer [or term used by patient]?

Moderator Note: *Ask questions 5.1 to 5.3 for the PROMIS item on global physical functioning items. Then continue to question 5.4.*

5.4. How important would it be for your liver cancer [or term used by patient] treatment to also have an impact on your daily physical activities? Why?

Moderator script: *We just discussed overall physical functioning. However, physical functioning has multiple aspects, which we would like to explore further with you.*

Moderator Note: *Ask questions 5.1 to 5.3 for all EORTC QLQ-C30 questions relating to physical functioning, and then for the PROMIS physical functioning items. Then continue to questions 5.5 to 5.11.*

5.5. How important would it be for your liver cancer [or term used by patient] treatment to also have an impact on your physical functioning? Why?

5.6. What effect on physical functioning would you be hoping for in a treatment? How big a change would be important to you?
PROBE:

- Ability to slow down worsening of your physical functioning
- Ability to improve physical functioning
- Ability to maintain your physical functioning at its current level
- Which aspect of physical functioning would you want the drug to address

Moderator Note: *show the physical functioning domain from EORTC QLQ-C30, "Physical functioning Set A"*

5.7. These questions are typically asked together. Together, do they cover all aspects of physical functioning to you?

5.8. Are all questions equally important to you for measuring your physical functioning?

Moderator Note: *Show the physical functioning items from the PROMIS item bank, "Physical functioning Set B"*

5.9. Another questionnaire asks these questions. Together, do they cover all aspects of physical functioning to you?

5.10. Are all questions equally important to you for measuring your physical functioning?

Moderator Note: *Show the physical functioning items from EORTC QLQ-C30 and the PROMIS item bank, "Physical functioning Set A and Set B"*

5.11. Do the questions from Set A sufficiently cover all aspects of physical functioning to you, or do you think the combination of Set A and Set B cover physical functioning better?

Moderator Note*: Show fatigue questions and ask questions 5.1 to 5.3 for all EORTC QLQ-C30 questions relating to fatigue. Once the patient has answered all the questions relating to fatigue, ask the following questions*

5.12. When you think of fatigue, what do you think about specifically? What other words come to mind? How would you describe your fatigue in greater detail?

PROBE

- Mental fatigue
- Physical fatigue

5.13. How important would it be for your treatment for your liver cancer [or term used by patient] to also treat your fatigue? Why?

5.14. What effect on fatigue would you be hoping for in a treatment? How big a change would be important to you?

PROBE:

- Ability to slow down worsening of your fatigue
- Ability to improve fatigue

- Ability to maintain your fatigue at its current level
- Which aspect of fatigue would you want the drug to address

Moderator Note: *Show the fatigue domain from EORTC QLQ-C30*

5.15. These questions are typically asked together. Together, do they cover all aspects of fatigue to you?

5.16. Are all questions equally important to you for measuring your fatigue?

Moderator Note: *Show the sleeping/problems/ insomnia question and ask questions 5.1 to 5.3**,** once the patient has answered the question on sleeping problems/ insomnia, ask the following questions*

5.17. Does this question cover all aspects of sleeping problems/ insomnia for you?

Moderator Note: *Show the role functioning (i.e. Interference in work, daily activities; hobbies or leisure activities) questions and ask questions 5.1 to 5.3**,** once the patient has answered the questions on role functioning, ask the following questions*

5.18. Do these questions cover all aspects of your functioning in all relevant settings to you (i.e. your family setting, your work, or your social circle(s)?

5.19. Are all questions equally important to you when it comes to how you function on a day to day basis?

Moderator script: *We would now like to go through another questionnaire with you, which was developed specifically for liver cancer [or term used by patient]. I would like you to answer the questions on the questionnaire based on **your current experience (i.e. in the past week)**.*

Moderator Note: *Set up questionnaire questions from EORTC QLQ-HCC18. Present questions one by one and allow the patient time to provide his or her answer to the question before moving to the next question (you may need to read the questions aloud and collect the patient answer, if so, please read the question exactly as they appear on the screen and do not provide any additional clarification). Please note that all EORTC QLQ-HCC18 questions have a recall period of 1 week, except for the last question, which has a recall period of 4 weeks.*

Moderator Note: *For questions with a recall period of 1 week*

5.20. What response would you choose based on your experience in the last week?

Moderator Note: *For questions with a recall period of 4 weeks*

5.21. What response would you choose based on your experience in the last 4 weeks?

Close (5 minutes)

Moderator script: *Thank you for this discussion. Your answers have been extremely valuable for this research. These are all the questions I have for you on this topic. Do you have any other comments or experiences you would like to share? (Allow the participant to provide any additional feedback). Thank you again for your time; this has been very helpful. To thank you for your time and effort today, we would like to compensate you with a $150 gift card. You can expect to receive this in the mail in 2-4 weeks.*

APPENDIX-1
HCC Signs and Symptoms (not exhaustive)

Categorization	*Symptoms*
GI	- **Constipation**<br>- **Diarrhea**<br>- **Vomiting**<br>- **Nausea**<br>- Flatulence<br>- Heartburn
Abdominal	- **Abdominal pain**<br>- **Abdominal swelling**
Skin	- Itchy skin<br>- Changes in skin<br>- Impaired wound healing<br>- Bruising
Sexual/ urogenital problems	- Sexual problems<br>- Reduced sexual interest<br>- Problem with urination
Extremities	- Swollen ankles/ arms / legs<br>- Numbness / tingling in hands and feet
Mouth and throat	- Cough / throat irritation / voice change<br>- Difficulty swallowing<br>- Change in taste<br>- Dry mouth<br>- Mouth sores
Other symptoms	- **Fatigue** / lack of energy / weakness<br>- **Sleep problem**<br>- **Jaundice**<br>- **Weight loss**<br>- **Appetite loss**<br>- Other pain, e.g. headache<br>- Dyspnea<br>- Dizziness<br>- Hair loss<br>- Fever / sweats<br>- Cold sensitivity<br>- Difficulty concentrating

Bolded symptoms were prioritized during the literature review

APPENDIX-2
HCC Impacts (not exhaustive)

Impacts
- **<u>Concern about appearance / body image</u>**<br>- **<u>Decreased role functioning (affected role at home)</u>**<br>- **<u>Decreased working ability</u>**<br>- **<u>Decreased ability to independently accomplish daily affairs / daily routine</u>**<br>- **<u>Sorrow / depression</u>**<br>- **<u>Anxiety / worry / nervousness</u>**<br>- **<u>Fear of death / panic</u>**<br>- **<u>Anger / irritability</u>**<br>- **<u>Social Isolation</u>**<br>- Decrease in exercise<br>- Frequent bed rest<br>- Decreased sense of satisfaction at work<br>- Decreased quality of life satisfaction<br>- Losing confidence in overcoming current disease<br>- Concern about disease progression<br>- Regret<br>- Difficulty concentrating

Bolded impacts were prioritized during the literature review
